# Left and right ventricular function in acutely presenting Amyocarditis: sparing of the right ventricle

**DOI:** 10.1186/1532-429X-13-S1-P331

**Published:** 2011-02-02

**Authors:** Shanti Velmurugan, Filip Zemrak, Neha Sekhri, Abdul Mozid, Redha Boubertakh, Vijay Anand, Francesca Pugliese, Anthony Mathur, Mark Westwood, Steffen Petersen, Ceri Davies, Saidi Mohiddin

**Affiliations:** 1 London Chest Hospital, London, UK

## Aim

To assess right ventricular (RV) function in patients with acute myocarditis.

## Background

Myocarditis is diagnosed in up to 10% of patients presenting as an acute coronary syndrome (ACS) who have elevated serum troponin and normal coronary angiogram. The diagnosis may be established by demonstrating typical patterns of abnormal tissue characterization at cardiac magnetic resonance imaging (CMR). Most reports examine left ventricular (LV) involvement; detecting tissue abnormalities in the right ventricle (RV) is limited by the technique’s spatial resolution. In patients diagnosed with myocarditis undergoing both acute and convalescent CMR scans, we studied changes in RV volumes to detect evidence of RV involvement.

## Methods

We studied acute and convalescent scans of 25 patients diagnosed with myocarditis following initial presentation with presumed ACS. Continuous short axis cine images covered the LV and RV (slice thickness 8mm, gap of 2mm, 1.5T Phillips Achieva). LV and RV end diastolic (LVEDV, RVEDV,) end systolic (LVESV, RVESV) and ejection fraction (LVEF, RVEF) were calculated (CMR 42, Circle Cardiovascular Imaging Inc, Calgary, Canada) and compared using the paired t test or the Wilcoxon signed rank test for skewed data.

## Results

Mean age was 39 ± 14 years, and 24% were female. Mean time to acute scan was 3 ± 3 days following admission (range 0-13 days). Mean time between convalescent scans was 13 ± 8 weeks (range 5-32 weeks). 24/25 patients had patchy LV delayed enhancement and/or myocardial oedema (T2 STIR) on the acute scan; these changes were not detected in the RV. LVEF (%) was lower in the acute scan compared to the convalescent scan: median 64 (IQR 59, 74) vs. 71 (IQR 63, 75; p<0.05). In contrast, RVEF (%) was unchanged: median 58 (IQR 54.5, 63) vs. 61 (IQR 54.5, 66; p=0.24). Mean LVEDV (mls) was 143 (SD ± 58) compared to 150 (SD ± 42; p=0.24). RVEDV (mls) was 156 (SD ± 58) compared to 160 (SD ± 44; p=0.53). Similarly, no significant differences were noted between the LVESV or RVESV.

## Conclusion

In this well characterized cohort of 25 patients, myocarditis is associated with changes in LV systolic function. Despite its thin walls, RV systolic function appears to be unaffected in acute presentations of myocarditis.

